# The future of global health education: training for equity in global health

**DOI:** 10.1186/s12909-016-0820-0

**Published:** 2016-11-21

**Authors:** Lisa V. Adams, Claire M. Wagner, Cameron T. Nutt, Agnes Binagwaho

**Affiliations:** 1 Center for Health Equity, Dartmouth’s Geisel School of Medicine, 1 Rope Ferry Road, Room 219, Hanover, NH 03755 USA; 2 Union for International Cancer Control, 62 Route de Frontenex, 1207 Geneva, Switzerland; 3 Department of Global Health and Social Medicine, Harvard Medical School, 641 Huntington Ave, Boston, MA 02115 USA; 4 University of Global Health Equity, Kigali, Rwanda

**Keywords:** Global health, Education, International

## Abstract

**Background:**

Among academic institutions in the United States, interest in global health has grown substantially: by the number of students seeking global health opportunities at all stages of training, and by the increase in institutional partnerships and newly established centers, institutes, and initiatives to house global health programs at undergraduate, public health and medical schools. Witnessing this remarkable growth should compel health educators to question whether the training and guidance that we provide to students today is appropriate, and whether it will be applicable in the next decade and beyond. Given that “global health” did not exist as an academic discipline in the United States 20 years ago, what can we expect it will look like 20 years from now and how can we prepare for that future?

**Discussion:**

Most clinicians and trainees today recognize the importance of true partnership and capacity building in both directions for successful international collaborations. The challenge is in the execution of these practices. There are projects around the world where this is occurring and equitable partnerships have been established. Based on our experience and observations of the current landscape of academic global health, we share a perspective on principles of engagement, highlighting instances where partnerships have thrived, and examples of where we, as a global community, have fallen short.

**Conclusions:**

As the world moves beyond the charity model of global health (and its colonial roots), it is evident that the issue underlying ethical global health practice is partnership and the pursuit of health equity. Thus, achieving equity in global health education and practice ought to be central to our mission as educators and advisors when preparing trainees for careers in this field. Seeking to eliminate health inequities wherever they are ingrained will reveal the injustices around the globe and in our own cities and towns.

## Background

In the United States (U.S.) and around the world, interest in global health as an academic discipline has skyrocketed over the last decade [1]. Students at all levels – sometimes as early as high school – are participating in projects and programs related to addressing health disparities outside of their home countries. More American students are entering medical school and residency programs having amassed a heterogeneous collection of overseas experiences, and are seeking advanced training in this budding field we now refer to as global health [2–5]. We find that many applicants inquire about institutional global health opportunities during their medical school interviews – a clear indication that global health offerings are at minimum an important factor in their school selection process. Increasing numbers of residency programs now offer global health tracks within or across specialties to accommodate trainees’ continued interest in and desire for clinical opportunities in global health [6–9]. Several non-profit organizations also offer volunteer service-learning opportunities, including structured community-based programs to educate students in global health. And yet, in spite of this demand-driven growth in the number of programs, breadth of opportunities, and amount of funding available to U.S. students, it has become apparent that our offerings do not consistently align with the priorities, needs, and preferences of our partners who generously host our trainees in the low- and middle-income settings that constitute the common global health destinations [10]. Furthermore, our reciprocity as hosts of students from under-resourced countries is often far from equitable [10].

In this article, we offer a perspective based on our review of the literature and collective experience of an emerging framework for global health opportunities for trainees rooted in equitable engagement, and present instances where such partnerships have thrived alongside examples of where we, as a global community, have fallen short. Although not an exhaustive assessment, our aim is to chronicle this particular moment in the history of global health training in the U.S., with the intent to help steer future directions towards best practices in training to promote equity in global health education and practice.

### Global health at academic institutions: a growth industry

As the field of global health emerges as its own academic and clinical discipline, medical schools in the U.S. are positioning themselves by establishing or strengthening related programs, centers, and institutes [11]. These new entities are often charged with defining curricular and co-curricular opportunities, and their leaders are contributing to the discussion of competencies in global health [12–14]. An intraprofessional education committee of the Consortium of Universities for Global Health is providing important guidance in this area [15]. A few schools are also taking a lead in the effort to disseminate this information without barriers, as exemplified by the freely available Global Health Delivery cases published by Harvard Business School, the open-access training modules by Unite for Sight, and all-access syllabi, readings, and taped lectures hosted by various U.S. universities or their open courseware partners such as EdX and Coursera. By broadening access, these new offerings are additionally helping to level the playing field in global health education [16]. In addition, a growing number of low- and middle-income countries, including (among many others) China, Thailand, Mexico, Rwanda, and South Africa, are now establishing their own global health education centers and institutions [17, 18].

Global health opportunities and career tracks appeal to faculty at all stages: from junior faculty who may have more lifestyle flexibility and recently acquired experience as students/trainees, to mid-career and retiring faculty (some with reinvigorated interest stemming from earlier overseas experiences) who seek to apply their skills in places strapped by the dual burdens of poverty and weak health systems. There are many entry points for global health work now at any stage in one’s career - from extended structured internships for students or trainees to shorter-term teaching or specialty practice opportunities for faculty. Good mentoring from an experienced global health practitioner can guide those new to the field on everything from funding sources to career pathways.

International research opportunities in both communicable and non-communicable diseases are on the rise, supported by numerous institutions including the Fogarty International Center, National Cancer Institute, National Institute of Allergy and Infectious Diseases, Grand Challenges Canada, and other major philanthropic initiatives such as the Gates Foundation, The Global Fund, and PEPFAR (President’s Emergency Plan for AIDS Relief). These opportunities have targeted clinical researchers as well as those engaged in global health training and technical assistance [19].

Witnessing this remarkable growth has many global health educators wondering whether the training and guidance that we provide to students today is appropriate, and whether it will be applicable in their future careers. Global health did not exist as an academic discipline when one of our authors was in medical school 25 years ago. What we now refer to as “global health” derives from an earlier discipline in academia called “international health,” itself preceded by the practice of “tropical medicine and hygiene” [20]. This evolution is largely documented in the changing of department and academic center names around the U.S., as well as in changes in scientific discourse around these issues, over the past three decades. It would have been difficult to predict the burgeoning interest in this area of study and practice over that time; nor could we have anticipated the resources now available to support the actual delivery of services through such partnerships. Further, as today’s global health practice has expanded to encompass the formal study of social justice and health equity, what indications do we have that we are teaching the right competencies and providing trainees with the right tools to tackle these issues in humble and effective ways?

## Discussion

### Recalibrating our efforts: a focus on health equity

Many who work in global health are driven by the same motive, aptly summarized as the desire “to work together towards a future in which *where* a patient lives doesn’t determine *if* they live” [21]. When queried, students, trainees and clinicians cite the opportunity to serve disadvantaged communities as a motivating factor for their decision to engage in global health and there are data to suggest global health exposure during training leads to careers in primary care and/or with underserved populations [22, 23]. While we may use different terms to describe our motivations – a sense of social justice, a moral or faith-based imperative, a sense of fairness, a belief in health as a human right or that all lives share equal value – the underlying commitment, in our experience, is usually founded in a desire to reduce or eliminate health inequities [24]. Indeed, one of the most widely cited definitions of global health published in the *Lancet* firmly puts health equity at the core of global health. Specifically, Koplan and his co-authors define global health as “an area for study, research, and practice that places a priority on improving health and achieving equity in health for all people worldwide [25].” If health equity is a common thread, we should be certain that our students have a strong foundation in understanding what health equity is, and how it can be achieved. As we are reminded throughout our work, the practice of global health begins wherever you put your feet. In other words, as with all systems of power, many health disparities are intensely local; trainees’ own communities are on the globe, too.

Definitions of health equity and inequity also abound, largely encompassing the concepts of systematic differences that result in worse health or greater health risks experienced by disadvantaged groups, often identified by social, racial, ethnic, economic or demographic distinctions [26, 27]. In recent decades, this area of inquiry has been well studied in a variety of settings, especially across different populations in high- and middle-income countries. Stark disparities of access and outcome persist in the shadows of some of the U.S.’s most renowned teaching hospitals: African-American women are one and a half times as likely to die of breast cancer than Caucasian women in Boston, while residents of the District of Columbia are 12 times more likely to die of HIV than their neighbors just across the border in Virginia [28, 29]. Policy research disaggregated by socioeconomic status, residence, gender, and ethnicity in poorer countries has enabled some national health systems to strive for and measure progress towards equitable access to medical advances. Similarly, by examining the cycle of service delivery, research and documentation, and policy and program adjustments that feed back into care delivery, overall implementation and evaluation practices can be improved. A few examples from sub-Saharan Africa (and specifically Rwanda, where we work) of this cycle of implementation science, policy uptake, and quality improvement are provided for further reading [30–35]. Like the global burden of disease and our collective commitment to lightening the load on those who bear its heaviest weight, global health pedagogy must be dynamic and versatile, with a focus on systemic solutions to address health disparities wherever they occur.

Since today’s challenges in global health are multifactorial and complex, our solutions must also be multidisciplinary. Today’s global health practitioners need to work effectively within interdisciplinary teams – alongside nurses and other allied health professionals, but also with educators, engineers, policy-makers, and industry representatives, to name a few. There must be an understanding of the various roles and contributions that colleagues across many disciplines can make. Working in multidisciplinary teams can be challenging, especially when different disciplinary perspectives do not align. This requires global health practitioners to develop the necessary skills to effectively support, nurture, and promote interdisciplinary thinking and approaches in their care teams. They must learn how to become what Rishi Manchanda calls, in his book by the same name, “upstream doctors” – providers who can contribute to a large team in healing the acute problem, while looking upstream to address the “causes of the causes” [36]. Eliminating or reducing healthcare inequities from the healthcare system may not guarantee the same healthcare outcomes for all, but it will certainly go a long way towards that goal.

### Towards true partnerships

Experts in global health recognize the importance of strong in-country collaborations in order to be effective, efficient, and responsive to the priorities of host countries and institutions. *How* to build effective partnerships has been the challenge. International health partnerships have historically been unbalanced, many times replicating past colonial relationships [37, 38]. Such collaborations have spanned deep chasms of inequality, and at times occasioned significant cognitive and moral dissonance on both sides of a relationship. One example of this double standard occurred at the turn of the millennium, at a time when some prominent American academics argued that antiretroviral therapy was too costly and too complex for Africans, yet American medical students traveled to Southern Africa on clinical electives in infectious disease with 30-day supplies of post-exposure prophylaxis for themselves.

American or European institutions have at times used teaching hospitals in low-income countries solely as sites at which to train their own students, or for their own faculty to conduct research without substantial engagement with or recognition of local health authorities and clinicians (so-called “parachute” research involving the extraction of patient data or samples and limited or non-existent capacity transfer) [39, 40]. While such blatant exploitation may be less common today, the reality is that variations of this practice do still occur, and there remains a subtle undercurrent of a superior/inferior dichotomy to many international collaborations. It is an unfortunate irony that work aimed at reducing inequities in health can sometimes replicate inequities in professional relationships.

While there is no single approach that applies to all international collaborations, there are common principles, values, and characteristics that form the foundation for effective, productive, and equitable partnerships. In Table 1, we summarize critical components or best practices for global health education and partnership based on our collective experience. These parameters can provide guidance to new or seasoned students, trainees, or faculty considering a particular global health project or program. Furthermore, early efforts to define useful metrics for exploring fairness in research and training collaborations are underway [41–43]. One group of researchers noted that there are four essential components to establishing and maintaining successful collaborations, these being: “1) mutual respect and benefit, 2) trust, 3) good communication, and 4) clear partner roles and expectations” [41]. One specific metric that some research consortiums employ or emphasize as a proxy for equitable partnerships (and one that some have made progress on) is the proportion of academic manuscripts led by host-country investigators [44, 45].

**Table 1:** Core components of equitable global health education and practice

1. Engagement of interdisciplinary teams and an ability for all global health practitioners to work respectfully and collaboratively
2. Development of equitable partnerships with shared leadership and stated, common goals
3. Alignment of priorities and research agendas that are driven by the low- or middle-income country partner
4. Program management, problem-solving, and where possible, financial oversight provided by the low- or middle-income partner
5. Education of trainees from the low- or middle-income country site is prioritized over education of trainees from the high-income country partner
6. Applications for research or programmatic funding opportunities are jointly conceived and written
7. Research conducted jointly with shared principal investigator and research team member roles, publication authorship and presentations, and broad availability of findings through publication in open-access or HINARI-supported journals

### A new architecture for partnership: developing best practices

Fortunately, today there are many examples of university partnerships where effective transfer of knowledge and skills occurs in both directions and where leadership is squarely in the hands of the host-country partner [46, 47]. One such example that we have been involved with is Rwanda’s Human Resources for Health (HRH) Program. Launched in 2012, this program is a comprehensive seven-year commitment to rebuild the Rwandan medical education system, with the overall goal of creating a sustainable, high-quality healthcare system. Eight medical schools, five nursing schools, two dental schools, and one health-management program comprised the initial consortium of U.S. partners sending faculty members to work in interdisciplinary teams alongside Rwandan partners at teaching hospitals across the country. This program has many unique features: the inclusion of multiple specialties and a wide range of health professions, the obliged lengthy duration of stay for the faculty (6–12 months for specialists and 2–3 months for subspecialists), and the number of collaborating schools, all of which have been described in detail elsewhere [48]. An important feature of this partnership that can be regarded as a foundational best practice in equitable engagement, is that the Rwandan Ministry of Health conceived of, advocated for, designed, implemented, and now manages this program.

A comprehensive memorandum of understanding outlines the relationships and responsibilities of each partner, but the U.S. partners serve at the invitation and under the direction of Rwandan leadership. In this relationship, American partners strive for Rwandan-developed and Rwandan-owned solutions to the medical education challenges the country faces two decades after the 1994 genocide that devastated its healthcare workforce. Priorities for health care delivery at the main teaching hospitals – from deciding whether to strengthen the pathology lab’s diagnostic capabilities first, or to instead begin with a focus on infection control – are decided not by funding opportunities or by which medical expert happens to be available, but by the Rwandan leadership based on their strategic vision. Measures of sustained success include demonstrable knowledge and skills among graduating trainees, numbers of trained physicians and nurses retrained in the Rwandan health sector and, ultimately, the impact on health indices for the population.

Ownership is critical to any national health system. While not every low-income country with a healthcare worker shortage may be in a position to pursue this type of program, we believe and have experienced how vital it is for the U.S. partners to *not* be at the helm, but rather accompany and/or partner with local authorities in pursuit of a shared vision.

Efforts like Rwanda’s HRH Program are well positioned to evolve into a meeting of equals: in this case, equal relationships in which each partner brings complementary skills and expertise to the table. Partnerships like this may also serve as excellent examples to young American students and trainees who wish to engage in global health work. Many of us who reside in better-resourced settings already know we have much to learn from our colleagues who work in settings of privation – not just about innovations in healthcare delivery, but also in the meaning of being allies toward a common set of goals. Stated another way, open minds and genuine humility are essential to two-way learning, both because brilliant ideas are born everywhere and because resource constraints have often led to creativity and novel methods with the potential to transform health systems in settings of all income levels. The notion of “reverse innovation” has been best described in the business literature [49] but this important concept of learning happening in both directions is now being applied to healthcare as well [50], including in Rwanda [51].

Another important best practice is a shared commitment to creating research and educational collaboratives in which each institution contributes in a unique and lasting way. If we expect these types of partnerships to become the norm in global health, we need to ensure that our students are well equipped to serve as good partners who know how to build equitable and successful collaborations across cultures, disciplines, and distances. Lists of required courses in global health may come to resemble those more common to a business school core curriculum – i.e., courses on negotiation and management – or the social science disciplines such as anthropology or sociology. A global health curriculum of the future will require flexibility, and will draw upon the expertise of many colleagues outside of the traditional basic and clinical sciences [52].

### Redefining roles

Beyond building effective and equitable partnerships, educators also need to consider appropriate roles for U.S.-trained global health practitioners of the future. As discussed, the past pattern of charitable assistance often has little or no role in the field of academic global health. Access to information is still a major bottleneck for many students in low-income countries, but less so with the advent of broadened Internet access and open-access scientific and medical journals (though these still constitute too small a proportion of the literature). The World Health Organization’s HINARI (Health InterNetwork Access to Research Initiative) Program, established with the support of major publishers, now provides students and faculty in low-income countries access to a wide range of electronic medical journals and other resources [53]. Broader access to original sources complements the previously mentioned growing array of courses, lectures, cases and other material freely available online.

The reality is that there are still – and may be for some years to come – sites in the world where there is either no clinician, or too few clinicians with sufficient training. While these positions will always be best filled by those who understand the culture and speak the language of the patients, there are some foreign doctors who make long-term commitments and assimilate as much as any outsider can, thus offering a real service to the population. It is likely there will continue to be trained clinicians in wealthier countries who will want to contribute through direct service delivery. As the recent Ebola epidemic in West Africa and the delayed international response and suboptimal coordination among U.S. academic medical centers in mobilizing clinical teams demonstrated, roles for skilled and committed health professionals to make critical contributions to global health – even for a relatively short duration – will likely remain for some time. In addition, until skill transfer is complete, there will always still be patients in low-resource areas who benefit from short-term visits by foreign surgical teams to perform procedures such as cleft palate repairs, obstetric fistula closures, and cardiac valve replacements [54].

Over the coming decade, as healthcare workforces overseas are strengthened further; as skill transfer to the rising generation of young, mobile, and tech-savvy healthcare professionals proceeds; and as our U.S. colleagues enter existing equitable partnerships or establish new ones, what role will the U.S. global health practitioner play, and how can we train the next generation to be ready for this role? The good news is that every specialty has a place on the global health stage (and we believe that every occupation can have a role to play – from architects to electricians to web designers and journalists – in promoting equity in global health). Most collaborations now cut across many, if not all, specialties, from dermatology to dentistry.

We know that the *true* experts on topics related to healthcare delivery in their own settings are the practitioners, policy-makers, clinicians, and patients themselves. And as such, U.S. global health trainees will need to learn competencies related to building partnerships in which they apply knowledge gained from their counterparts and continue to add value. Careful self-evaluation of this nature is emerging as an important priority for practical inquiry in global health practice. Kolars and colleagues have described criteria to assess international medical school partnerships to provide guidance in this area [55].

From our experience, we do believe that the majority of students share this desire to achieve health equity for all. While the term “health equity” does not yet generate the same buzz as the concept of “global health” does among today’s students, we predict that someday it will. But this change of mindset will not occur on its own. It will require an unwavering commitment to achieving equity, to striving for reciprocity in opportunities for training and career development, and to reaching a shared understanding of what constitutes sustainable change among all partners. Additionally, it must come with the acknowledgment in action and words that we have tremendous inequities in the U.S. as well, and that work in health equity really does start wherever you put your feet.

Thus, if we want our students to be versatile to practice wherever there are health inequities, we should reorient their training accordingly to include the necessary skillset for modern anti-colonialist global health practice, and to emphasize best practices of equity in health education and practice. We believe there are more similarities than differences in working with underserved populations who have been subjected to systematic health inequities, regardless of whether they live in rural New Hampshire or urban Dar es Salaam. As The World Bank did recently when it restructured its programs to be centered on “global practices” instead of regions, we need to think similarly about health equity issues being applicable across contexts, and about connections that are important for trainees to understand. In other words, we need to teach our students to recognize these similarities, and equip them to take on the challenges, regardless of the setting. Looking to the future, we predict that such training will generate the most effective global health practitioners – some of whom may opt to practice in underserved U.S. communities where stark needs exist, too.

## Conclusions

As we move beyond the outdated “North-assists-South” model in global health, we must adapt the roles of our global health specialists and adjust their training accordingly, with increased focus on equitable partnership development. In the present article, we aimed to provide one perspective on essential aspects of global health training for students in U.S. academic institutions based on our collective experiences and a review of the medical education literature. Realizing that a desire for health equity is at the heart of most global health work provides one direction. Challenging students and trainees in the U.S. to focus on health equity as the underlying and unifying issue should prepare them well for a vocation dedicated to the pursuit of justice through health, regardless of location.

## Abbreviations

HINARI: Health InterNetwork Access to Research Initiative
HRH: Human Resources for Health
PEPFAR: President’s Emergency Plan for AIDS Relief
U.S.: United States
